# At What Age to Operate for Squint

**Published:** 1899-09-30

**Authors:** 


### AT WHAT AGE TO OPERATE FOR SQUINT.

The very important address on the treatment of strabismus in young children, delivered by Professor Priestley Smith before the International Ophthalmic Congress at Utrecht, dealt with a question in regard to which it is very desirable that practitioners should possess clear ideas, so that they shall be able to speak confidently in recommending a line of treatment which may not always be very acceptable to parents. In considering the 576 cases of concomitant convergent strabismus tabulated by the author of the address the first thing to be noted is that in 60 per cent. of them the strabismus began before the children were four years old; that arranging the cases in biennial groups the chief period of onset is that which includes children of two and three years old, and that more cases begin at three years old than at any other age. Then it is to be noted how long a delay took place in the majority of cases before the children were brought for treatment, a delay which was sometimes due to neglect and carelessness on the part of the parents, but more often to caution and to a reluctance to submit their children to definite treatment, which is perhaps not unnatural in view of the facts that on the one hand some cases appear to recover without any treatment, while on the other, if treatment is to be undertaken, even medical men do not seem to be of one mind as to how and when it is best to interfere. The third point to be noted is the harm which arises from delay. The most obvious of the visual defects met with among those who squint is the loss of fixation power, the inability to "fix" properly with the squinting eye even when the good eye is covered. Fixation power should be tested in the dark room with the help of the ophthalmoscope. The good eye is well covered, the light from the mirror is thrown on the squinting eye, and the child's attention is directed to it. The position of the corneal reflex in the illuminated pupil tells the observer whether fixation is true or false. False is found only in a minority of cases, and among the causes of its occurrence there are two which are certain, namely, early onset and long duration. The real question, however, in which so much of the treatment hinges is this, Is it the early squint that impairs the faculty of fixation, or is it an initial absence of fixation power that leads to early squint? In the main it is the former, for among the cases analysed no child was found to have false fixation who had not squinted for at least six months. Clearly it is largely a question of duration. In regard to this point it must be remembered that the development of vision is a gradual process extending over several years, and fixation has no stability at first. This is a faculty which is easily unlearned, and easily learned in a perverted form, and by long continuance the perverted form becomes ingrained and rendered permanent. But there is much reason to believe that an eye which is thrown out of use before its functional development is complete is likely to remain undeveloped and defective, so that not only does long duration appear to be a cause of loss of fixation power, but must also be credited with some of the secondary results of squint which may be classed as those arising from non-use or perverted function. In regard to treatment Professor Priestley Smith follows the logical teaching of the facts, and would begin early. Not that by the word treatment operation is necessarily meant, but he would not allow a child to drift into losing its fixation power in deference to the idea that nothing can be done at an early age. First, then, a complete examination should be made, an examination which must be entirely objective, and must include an estimation of the refraction. Then the means of treatment are, broadly speaking, three: (1) The diminution of accommodative effort and the sharpening of vision by glasses, and, if necessary, the suspension of acommodation by the continued use of atropine; (2) exercise of the squinting eye, or of both eyes alternately, by the use of a pad or shade; and (3) tenotomy. It is in regard to the latter that the question chiefly arises. It must be understood that late operations sometimes give perfect cures. But there are many cures so-called in which the eyes "fix," but in which true binocular vision is never reacquired, and the question is whether the number of these might not be diminished by operating earlier. It may then be said that while in every case an effort should be made to do without operation, and while the treatment adopted with this object should be continued so long as improvement continues to occur under its use, yet when progress comes definitely to a standstill and operation offers the only prospect of binocular vision, then it is right to operate, especially if the child be young. Professor Priestley Smith has operated several times at two years old, and more frequently at three and four years, and has seen no reason to regret it.

